# Using radiomics model for predicting extraprostatic extension with PSMA PET/CT studies: a comparative study with the Mehralivand grading system

**DOI:** 10.1186/s40644-025-00894-w

**Published:** 2025-06-18

**Authors:** Linjie Bian, Fanxuan Liu, Yige Peng, Xinyu Liu, Panli Li, Qiufang Liu, Lei Bi, Shaoli Song

**Affiliations:** 1 Department of Nuclear Medicine, Fudan University Shanghai Cancer Center, Shanghai, China; 2 Institute of Translational Medicine, National Center for Translational Medicine, Shanghai Jiao Tong University, Shanghai, China; 3 Shanghai Engineering Research Center of Molecular Imaging Probes, Shanghai, China; 4 Department of Oncology, Shanghai Medical College, Fudan University, Shanghai, China; 5 Center for Biomedical Imaging, Fudan University, Shanghai, China; 6 Key Laboratory of Nuclear Physics and Ion-beam Application (MOE), Fudan University, Shanghai, China; 7 College of Chemistry and Materials Science, Shanghai Normal University, Shanghai, China; 8 Department of Radiology, Wuxi No.2 People’s Hospital, Wuxi, Jiangsu China

**Keywords:** PSMA PET/CT, Radiomics, Extraprostatic extension (EPE)

## Abstract

**Purpose:**

This study aimed to evaluate the effectiveness of using a radiomics model to predict extraprostatic extension (EPE) in prostate cancer from PSMA PET/CT, and to directly compare its performance with the Mehralivand Grading System, an MRI-based method for EPE assessment.

**Methods:**

A total of 206 patients who underwent radical prostatectomy were included in this study. Radiomics features were extracted from PSMA PET/CT images to construct predictive models using Support Vector Machine (SVM) and Random Forest algorithms. In addition, among the 63 patients who underwent both PSMA PET/CT and multiparametric MRI (mpMRI), the performance of the radiomics model was compared with that of the Mehralivand Grading System. Key performance metrics, including the area under the curve (AUC), sensitivity, specificity, positive predictive value (PPV), and negative predictive value (NPV), were reported.

**Results:**

Among the 63 patients who underwent both PSMA PET/CT and multiparametric MRI (mpMRI), the radiomics model achieved an AUC of 76.8% (95% CI: 64.4–86.5%), sensitivity of 72.0%, specificity of 81.5%, PPV of 72.0%, and NPV of 81.6%. In comparison, the Mehralivand Grading System yielded AUCs of 66.8%, 63.5%, and 60.2% from three independent readers. DeLong’s test showed that the radiomics model significantly outperformed all three readers in terms of AUC (*p* = 0.013, 0.003, and 0.001, respectively).

**Conclusion:**

The radiomics model derived from PSMA PET/CT can better capture features associated with EPE and shows promise for aiding preoperative assessment in prostate cancer. However, further validation in larger, independent cohorts is necessary to confirm its stability and clinical utility.

**Supplementary Information:**

The online version contains supplementary material available at 10.1186/s40644-025-00894-w.

## Introduction

Radical prostatectomy (RP) is the primary treatment for localized prostate cancer [1]; however it is associated with a significant incidence of complications, including urinary incontinence and erectile dysfunction, with rates as high as 40.8–64% [2]. Nerve-sparing (NS) techniques have been developed to help to preserve urinary and sexual function postoperatively [3]. However, when extraprostatic extension (EPE) is present, NS techniques may increase the risk of positive surgical margins and biochemical recurrence. Therefore, accurately predicting EPE is crucial for tailoring surgical strategies [4].

Since the introduction of ^68^Ga-PSMA-11 in 2012 for prostate cancer imaging, tracers targeting prostate-specific membrane antigen (PSMA) have become a focal point in nuclear medicine research [5]. Although PSMA PET/CT has shown excellent performance in diagnosing and staging (N and M stages) of prostate cancer, however, due to the low soft-tissue resolution of CT, the accuracy of PSMA PET/CT shows lower accuracy in T-staging and EPE assessment when compared to mpMRI and PSMA PET/MR [6, 7]. Compared to PET/MR or multiparametric MRI (mpMRI), PET/CT presents several advantages, including broader availability and accessibility, more rapid imaging processes, lower associated costs, and suitable for patients with metal implants or pacemakers [8]. These factors collectively make PSMA PET/CT a more practical, cost-effective, and versatile option in clinical practice. Hence, developing non-invasive methods using PSMA PET/CT to accurately predict EPE is critical for enhancing diagnostic accuracy and optimizing clinical management.

Radiomics, a rapidly advancing field, involves converting medical images into high-dimensional quantitative features such that enables the extraction of information imperceptible to the naked eye through computational algorithms [9]. This approach improves lesion classification and risk stratification. Currently, numerous studies have demonstrated that radiomics offers a non-invasive and effective method for predicting EPE [10–12]. Despite these advancements, the application of radiomics to PSMA PET/CT, particularly in the context of EPE prediction, remains underexplored. Most existing studies on radiomics in prostate cancer have focused on mpMRI, leaving a gap in understanding how PSMA PET/CT-based radiomics can be optimized for EPE assessment [13]. In this study, we developed a radiomics model based on PSMA PET/CT and, for the first time, conducted a head-to-head comparison with the Mehralivand Grading System [14], an MRI-based method for EPE prediction, providing a novel perspective on optimizing surgical planning for prostate cancer patients.

## Materials and methods

### Study design

**Fig. 1:**
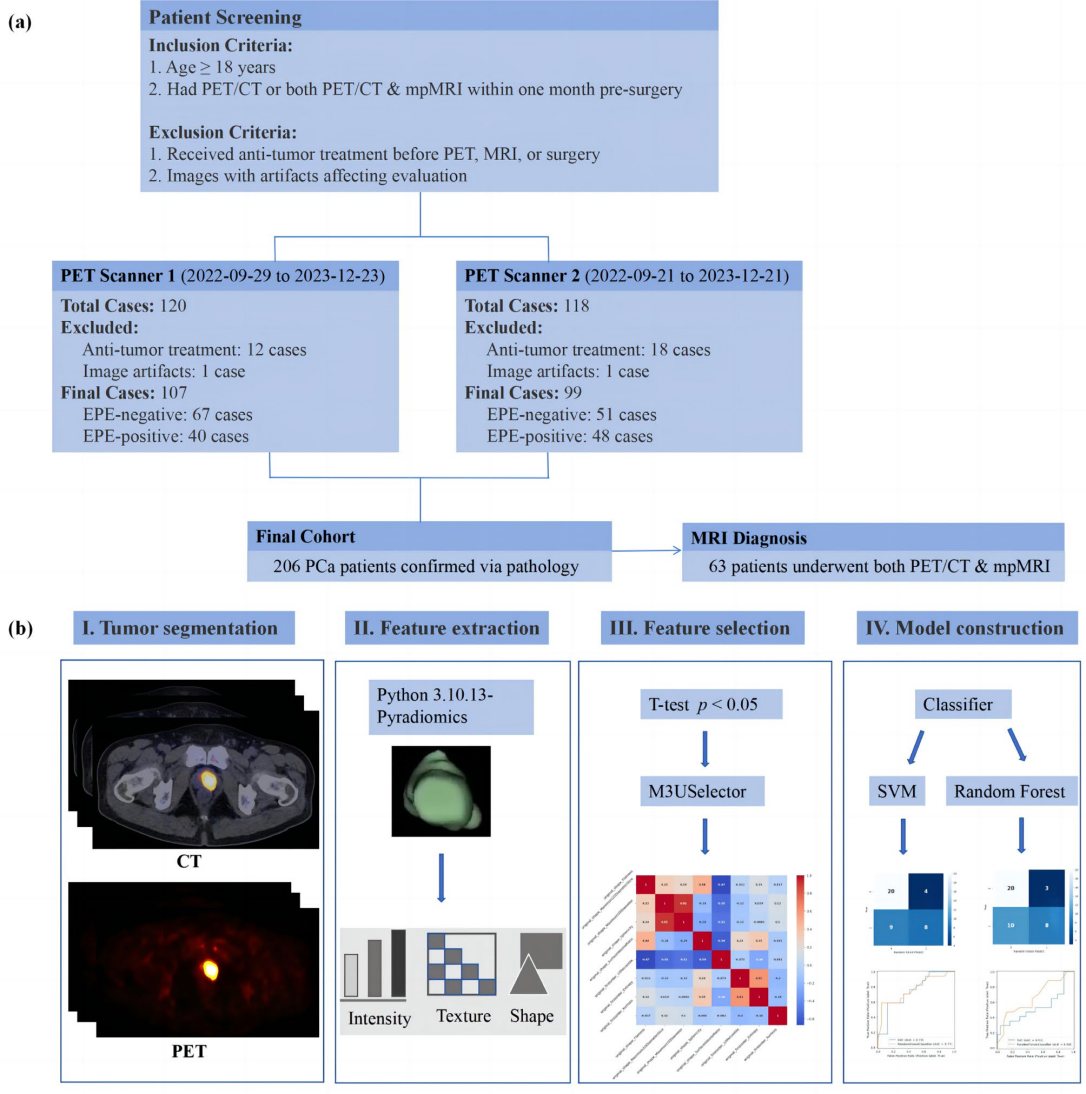
This figure consists of two panels: (**a**) presents the flowchart outlining the process of patient enrollment, and (**b**) depicts the overall study design

The study was approved by the Ethics Committee of Fudan University Shanghai Cancer Center (Ethical approval number: 1612167-18). This study included patients who underwent radical prostatectomy (RP) between September 2022 and December 2023. The inclusion criteria were: [1] Age ≥ 18 years; [2] Underwent an ^18^F-PSMA-1007 PET/CT scan within one month before surgery, or both an ^18^F-PSMA-1007 PET/CT and mpMRI scan. T4he exclusion criteria were: [1] Received anti-tumor treatment before the ^18^F-PSMA-1007 PET, MRI scan, or surgery; [2] Images with artifacts that affected evaluation. Ultimately, 206 patients were recruited, of which 107 cases used PET scanner 1 (Siemens CTI RDS Eclips ST, Knoxville, Tennessee, USA) and 99 cases used PET scanner 2 (United Imaging uMI 780, Shanghai, China) (Fig. 1a). The overall study design is shown in Fig. 1b.

### Clinical data collection

Detailed demographic and clinical data within two weeks prior to surgery, including age, BMI, and tumor markers, were obtained from electronic medical records. Pathological data were sourced from pathology reports and included biopsy Gleason scores, International Society of Urological Pathology (ISUP) grade groups, and whether there was extraprostatic extension (EPE) in the postoperative pathology.

### MRI imaging technique and MRI evaluation

Details of the imaging techniques are provided in Appendix E1. Three readers (Reader 1: 10 years of experience; Reader 2: 8 years of experience; Reader 3: 2 years of experience) independently assessed the EPE status of all enrolled patients based on bpMRI images, without knowledge of the pathological results. The assessments were primarily based on T2WI images. DWI and ADC images were used as auxiliary tools to delineate the boundaries of the lesion and the prostatic capsule. If a patient had multiple lesions, each lesion’s EPE status was evaluated separately, and the highest EPE score among the lesions was selected as the patient’s final score. Detailed Mehralivand grading criteria can be found in Appendix E2.

### Model construction and assessment

For detailed information on PSMA-PET/CT imaging specifics, PSMA-PET/CT image preprocessing, and feature extraction, please refer to Appendix E3. PET, CT and PET/CT fusion hand-crafted radiomic features (HRFs) are then selected and used for prediction. The PET/CT fusion features can be obtained by feature concatenation and feature averaging. The feature concatenation method (PET/CT-concat) combined the CT feature set and PET feature set of the same patient into a single feature vector by concatenating them. The feature averaging method (PET/CT-mean) added the PET and CT feature values of the same patient and then averaged the feature values. In this project, we used the T-test (ttest_ind function in the Python statsmodels package in Scipy 1.12.0) for feature selection (significance difference *p* < 0.05). We further refined the features with significant differences using the M3USelector in scikit-learn 1.4.0, retaining those features that contribute the most to the model’s performance.

Different machine learning classifiers, namely support vector machine (SVM), and Random Forest, were investigated for the prediction of EPE. The scikit-learn 1.4.0 package in Python 3.10.13 was used for building machine learning models. Five-fold stratified cross-validation (CV) was implemented, i.e., the dataset was split into five distinct subsets (folds), ensuring that each fold maintains the same proportion of class labels as the original dataset, in each iteration, one fold (20%) is used for validation and the other four (80%) are used for training e.g., building the models and tuning the hyperparameters. Subsequently, the performance of the trained model was evaluated on the validation data. The average performance of the classifiers across the 5 folds in the validation cohort served as the estimate of the CV estimators.

### Statistical analyses

All statistical analyses were conducted using SPSS (version 25.0, IBM Corporation, Armonk, USA) and R software (version 3.4.1, R Foundation for Statistical Computing, Vienna, Austria). To evaluate the predictive performance of the two methods (radiomics model and radiologist assessment), both were analyzed in terms of the area under the ROC curve (AUC), accuracy, sensitivity, specificity, positive predictive value (PPV), and negative predictive value (NPV). To compare the AUCs between the radiomics model and each radiologist, the DeLong test was used, as implemented in the R package pROC. A two-sided p-value < 0.05 was considered statistically significant.

## Results

### Baseline characteristics

This study included a total of 206 patients with pathologically confirmed prostate cancer (PCa) after radical surgery (88 with EPE-positive and 118 with EPE-negative). Among them, 63 patients underwent both ^18^F-PSMA-1007 PET/CT and mpMRI (25 with EPE-positive and 38 with EPE-negative). Detailed information on patient age, BMI, PSA levels, Biopsy ISUP grade, and positive biopsies can be found in Table S1.

### Performance of the radiomics models

**Fig. 2:**
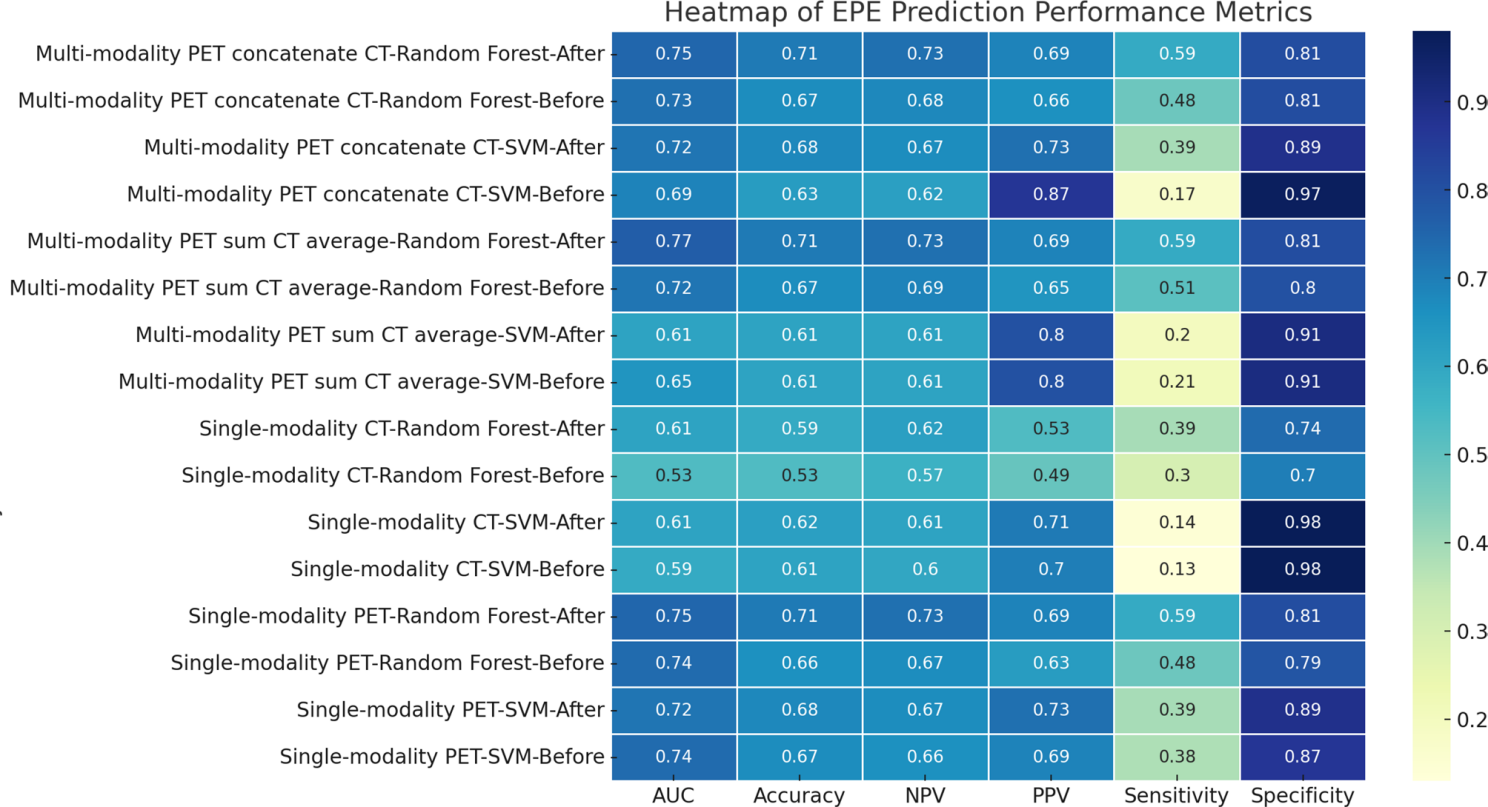
This figure is a heatmap showing the performance metrics (Accuracy, AUC, Specificity, Sensitivity, NPV, and PPV) across different modalities, classifiers, and feature selections

Details of the feature selection process and the specific features retained for each modality are provided in Appendix E4. We evaluated the performance of two machine learning classifiers, Support Vector Machine (SVM) and Random Forest, in predicting EPE across different modalities (Table 1; Fig. 2). Detailed Experimental Results are provided in Appendix E5.

#### Single-modality PET and CT features

PET Features: Before feature selection, the Random Forest model and the SVM model performed similarly with PET features. The former achieved an average AUC of 0.74, accuracy of 0.66, specificity of 0.79, sensitivity of 0.48, NPV of 0.67, and PPV of 0.63, while the SVM model had an average AUC of 0.74, accuracy of 0.67, specificity of 0.87, sensitivity of 0.38, NPV of 0.66, and PPV of 0.69. After feature selection, the Random Forest improved in AUC (0.75), accuracy (0.71) specificity (0.81), sensitivity (0.59), NPV (0.73), and PPV (0.69). However, the AUC of the SVM model slightly decreased to 0.72, while other metrics of the SVM model slightly improved with an accuracy of 0.68, specificity of 0.89, sensitivity of 0.39, NPV of 0.67, and PPV of 0.73.

CT Features: SVM model outperformed the Random Forest model before and after feature selection processes. After feature selection, the SVM model’s average AUC improved from 0.59 to 0.61, accuracy increased from 0.61 to 0.62, specificity remained at 0.98, sensitivity increased from 0.13 to 0.14, NPV increased from 0.60 to 0.61 and PPV remained at 0.70. In contrast, Random Forest model’s AUC increased from 0.53 to 0.61, accuracy improved from 0.53 to 0.59, specificity increased from 0.70 to 0.74, sensitivity increased from 0.30 to 0.39, NPV increased from 0.57 to 0.62, PPV increased from 0.49 to 0.53.

#### Multi-modality PET and CT features

Concatenated PET and CT Features: After concatenating PET and CT features, the performance of both SVM model and Random Forest model improved. After feature selection, the SVM model’s average AUC increased from 0.69 to 0.72, accuracy improved from 0.63 to 0.68, sensitivity increased from 0.17 to 0.39, NPV increased from 0.62 to 0.67, while specificity decreased from 0.97 to 0.89, PPV decreased from 0.87 to 0.73. The Random Forest model’s average AUC increased from 0.73 to 0.75, accuracy improved from 0.67 to 0.71, specificity remained at 0.81, sensitivity increased from 0.48 to 0.59, NPV increased from 0.68 to 0.73, PPV increased from 0.66 to 0.69.

Averaged PET and CT Features: After averaging PET and CT features, Random Forest model outperformed the SVM model. After feature selection, the Random Forest model achieved an average AUC of 0.77, accuracy of 0.71, specificity of 0.81, sensitivity of 0.59, NPV of 0.73, and PPV of 0.69, while the SVM model had an average AUC of 0.61, accuracy of 0.61, specificity of 0.91, sensitivity of 0.20, NPV of 0.61, and PPV of 0.80.

**Table 1:** EPE prediction performance across different modalities and classifiers

Modality	Feature Selection	Classifier	Accuracy	AUC	Specificity	Sensitivity	NPV	PPV
Single-modality PET	Before	SVM	0.67	0.74	0.87	0.38	0.66	0.69
Single-modality PET	After	SVM	0.68	0.72	0.89	0.39	0.67	0.73
Single-modality PET	Before	Random Forest	0.66	0.74	0.79	0.48	0.67	0.63
Single-modality PET	After	Random Forest	0.71	0.75	0.81	0.59	0.73	0.69
Single-modality CT	Before	SVM	0.61	0.59	0.98	0.13	0.60	0.70
Single-modality CT	After	SVM	0.62	0.61	0.98	0.14	0.61	0.71
Single-modality CT	Before	Random Forest	0.53	0.53	0.70	0.30	0.57	0.49
Single-modality CT	After	Random Forest	0.59	0.61	0.74	0.39	0.62	0.53
Multi-modality PET concatenate CT	Before	SVM	0.63	0.69	0.97	0.17	0.62	0.87
Multi-modality PET concatenate CT	After	SVM	0.68	0.72	0.89	0.39	0.67	0.73
Multi-modality PET concatenate CT	Before	Random Forest	0.67	0.73	0.81	0.48	0.68	0.66
Multi-modality PET concatenate CT	After	Random Forest	0.71	0.75	0.81	0.59	0.73	0.69
Multi-modality PET sum CT average	Before	SVM	0.61	0.65	0.91	0.21	0.61	0.80
Multi-modality PET sum CT average	After	SVM	0.61	0.61	0.91	0.20	0.61	0.80
Multi-modality PET sum CT average	Before	Random Forest	0.67	0.72	0.80	0.51	0.69	0.65
Multi-modality PET sum CT average	After	Random Forest	0.71	0.77	0.81	0.59	0.73	0.69

PET = positron emission tomography; SVM = support vector machine

Note.— AUC = area under the receiver operating characteristic curve, NPV = negative predictive value, PPV = positive predictive value

### Performance of three independent readers

In this study, three readers independently assessed the extracapsular extension (EPE) of peripheral lesions using the Mehralivand grading system and analyzed its diagnostic performance.

Table 2 presents the diagnostic performance of three readers in assessing EPE using the Mehralivand grading system. The results show that Reader 1 had an AUC of 66.8%, sensitivity of 60.0%, specificity of 73.7%, accuracy of 68.3%, PPV of 60.0%, and NPV of 73.7%. Reader 2 had an AUC of 63.5%, sensitivity of 56.0%, specificity of 71.1%, accuracy of 65.1%, PPV of 56.0%, and NPV of 71.1%. Reader 3 had an AUC of 60.2%, sensitivity of 52.0%, specificity of 68.4%, accuracy of 61.9%, PPV of 52.0%, and NPV of 68.4%.

Table S2 further analyzes the positive predictive value (PPV) for different Mehralivand grades. For grade 0, the PPVs for the three readers were 73.6%, 71.1%, and 68.4%, respectively; for grade 1, the PPVs were 47.1%, 44.4%, and 42.1%; for grade 2, the PPVs were 85.7%, 83.3%, and 80.90%; and for grade 3, the PPV was 100% for all three readers.

**Table 2:** Diagnostic performance of three readers and the radiomics model in assessing EPE, with AUC comparisons using delong’s test

	AUC(%)	Sensitivity (%)	Specificity (%)	Accuracy (%)	PPV (%)	NPV (%)	DeLong *P*-value (vs. Radiomics)
Reader 1	66.8 (53.8,78.2)	60 (38.7, 78.9) [15/25]	73.7 (56.9, 86.6) [28/38]	68.3 (55.3, 79.4) [43/63]	60 (44.6, 73.6) [15/25]	73.7 (62.6,82.4) [28/38]	0.013
Reader 2	63.5 (50.4,75.3)	56.0 (34.9,75.6) [14/25]	71.1 (54.1, 84.6) [27/38]	65.1 (52.0, 76.7) [41/63]	56 (40.9, 70.0) [14/25]	71.1 (60.1, 80) [27/38]	0.003
Reader 3	60.2 (51.2,69.3)	52.0 (31.3, 72.2) [13/25]	68.4 (51.3, 82.5) [26/38]	61.9 (48.8, 73.9) [39/63]	52 (37.3, 66.4) [13/25]	68.4 (57.7,77.5) [26/38]	0.001
Radiomics	76.8 (64.4,86.5)	72 (50.6, 87.9) [18/25]	81.5 (65.7, 92.3) [31/38]	77.8 (65.5, 87.3) [49/63]	72 (55.8, 84.0) [18/25]	81.6 (69.9,89.4) [31/38]	

Note.—Data in parentheses are 95% CIs, and data in brackets are numbers of patients. NPV = negative predictive value, PPV = positive predictive value

### Comparison of radiomics model performance with radiologist performance

To directly compare radiomics-based prediction with conventional MRI evaluation, a subset of 63 patients who underwent both ^18^F-PSMA-1007 PET/CT and mpMRI was selected from the full cohort. The radiomics model, developed using the full dataset of 206 patients, was applied to this subset. Using the radiomics model constructed with averaged PET and CT features and a Random Forest classifier, the model achieved an AUC of 76.8% (95% CI: 64.4%, 86.5%), sensitivity of 72.0%, specificity of 81.5%, accuracy of 77.8%, PPV of 72.0%, and NPV of 81.6%.

In contrast, three radiologists evaluated the same 63 cases using the Mehralivand grading system on mpMRI. Their AUCs were 66.8%, 63.5%, and 60.2%, respectively. DeLong’s test showed that the radiomics model significantly outperformed all three readers, with p-values of 0.013, 0.003, and 0.001, respectively (Table 2). These findings suggest that PSMA PET/CT-based radiomics provides superior diagnostic performance in predicting EPE compared to MRI-based visual assessment.

## Discussion

This study compares the performance of radiomics models using PSMA PET/CT with the Mehralivand grading system in predicting extracapsular extension (EPE) in prostate cancer, which underlines the diagnostic advantages of radiomics when combined with molecular imaging. The traditional Mehralivand grading system, however, exhibits certain limitations in assessing grades 0 and 1.

In this study, both the SVM and Random Forest models demonstrated good diagnostic performance, outperforming the Mehralivand grading system,. This indicates that radiomics models are more effective in identifying key features of EPE in prostate cancer. Support Vector Machine (SVM) and Random Forest are commonly used machine learning classification algorithms. SVM excels in high-dimensional spaces, particularly in handling nonlinear classification problems through the use of kernel functions [15–17]. Random Forest, on the other hand, enhances model robustness and generalization by constructing multiple decision trees, effectively reducing overfitting [18–20]. Both algorithms support feature selection and importance ranking, making radiomics models more precise and comprehensive in predicting EPE in prostate cancer.

We anticipate that PSMA PET/CT, targeting the highly expressed PSMA in prostate cancer, can identify invasive biomarkers at an earlier stage and more accurately [21–23]. This characteristic gives PSMA PET/CT a unique advantage in predicting EPE, especially in lesions that are difficult to discern with traditional imaging, showing higher sensitivity. Other radiomics models are often based on conventional MRI imaging, which, while providing good soft tissue contrast, lack the molecular precision of PSMA PET/CT.

**Fig. 3:**
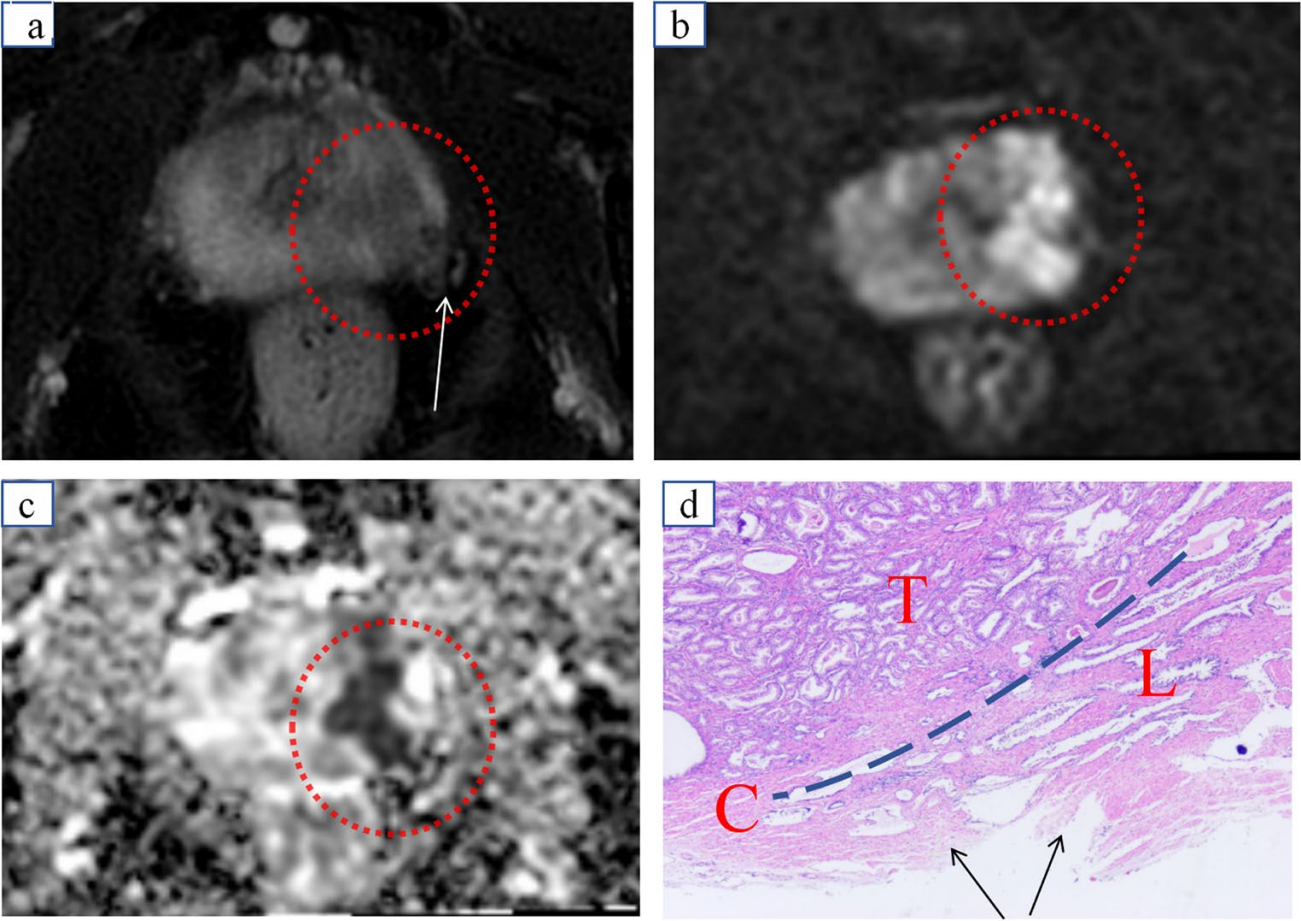
A typical case of false-positive misdiagnosis of EPE. The patient is a 72-year-old male. (**a**) T2WI shows a hypointense nodule in the left peripheral zone of the prostate (red circle) with a fibrous band shadow outside the prostatic capsule (white arrow); (**b**) DWI reveals a hyperintense signal (red circle); (**c**) ADC shows decreased values (red circle). The MRI diagnosis was prostate cancer. However, due to the interference of inflammation in the evaluation of the local prostatic capsule morphology, it was misjudged as EPE grade 1, leading to a diagnostic error. (**d**) Pathology: T represents the tumor, C and the blue curve indicate the prostatic capsule, the black arrow points to the pathological fibrotic focus, and L represents lymphocytes (HE *20)

Although the Mehralivand grading system shows higher accuracy in diagnosing EPE at higher grades (such as grades 2 and 3), its performance is relatively poor in diagnosing lower grades (such as grades 0 and 1), with significant variability in results among physicians. This subjectivity may stem from the reliance on the physician’s experience and judgment during the evaluation process, where slight differences in lesion and capsular contact in lower-grade EPE may lead to inconsistent standards among physicians, thus affecting the reliability of the diagnosis. Our follow-up revealed that the reason for the low positive predictive value (PPV) in grade 1 EPE was due to the local capsular morphology of the prostate often being affected by pathophysiological changes such as inflammation, puncture injuries, and connective tissue hyperplasia [24–26], which led to EPE grade 0 being misjudged as grade 1, resulting in false positives, as illustrated in Fig. 3.

However, this study has certain limitations. Due to the relatively limited sample size, the generalizability of the radiomics model needs to be further validated in larger independent cohorts. In future research, we will leverage larger datasets to strengthen the model’s robustness. Moreover, we plan to enhance the model using deep learning algorithms to capture more complex patterns in the imaging data. By integrating imaging data, clinical information, and radiomic features, we aim to develop advanced multimodal machine learning and deep learning models that can further improve predictive performance.

## Conclusion

This study suggests that radiomics models based on PSMA PET/CT may offer improved performance over the traditional Mehralivand grading system in predicting extracapsular extension (EPE) in prostate cancer. The potential advantage of the radiomics approach lies in the incorporation of molecular imaging features, which may help detect subtle lesion characteristics that conventional MRI assessments could miss. These findings highlight the promise of PSMA PET/CT radiomics for enhancing preoperative assessment and informing clinical decision-making. Nevertheless, due to the limited number of patients in certain subgroups, particularly those with lower Mehralivand grades (grades 0 and 1), subgroup analysis was not feasible in this study. Future validation in larger, independent cohorts is essential to confirm the model’s robustness across different grades and to further improve its clinical applicability.

## Electronic supplementary material

Below is the link to the electronic supplementary material.

Supplementary Material 1

## Data Availability

No datasets were generated or analysed during the current study.

## Abbreviations

PSMA: Prostate-specific membrane antigen
EPE: Extraprostatic extension
SVM: Support Vector Machine
RP: Radical prostatectomy
NS: Nerve-sparing
