# Supplementary material for: Using radiomics model for predicting extraprostatic extension with PSMA PET/CT studies: a comparative study with the Mehralivand grading system
Source: Cancer Imaging. 2025 Jun 18;25:77. doi: 10.1186/s40644-025-00894-w (PMC12177976; doi:10.1186/s40644-025-00894-w)
Supplement: Supplementary file 1 — Supplementary Material 1 [file 40644_2025_894_MOESM1_ESM.docx]

***Supplementary material***

**Appendix E1: MRI Imaging Technique**

Preoperative MRI was performed on two 3.0-T MRI systems (MAGNETOM Skyra 3.0 T, Siemens Healthineers, Erlangen, Germany). The entire prostate gland and seminal vesicles were imaged on coronal, sagittal, and axial slices using T2WI, DWI, and DCE. T2WI was completed with a fast-recovery fast-spin-echo (FR-FSE) sequence [repetition time (TR)/echo time (TE), 7,120 ms/89 ms; number of excitations, 2; slice thickness, 3 mm; spacing, 1 mm; matrix 324×320]. T1WI was completed with a fast spoiled gradient-echo (FSPGR) sequence (TR/TE, 231 ms/2.5 ms; slice thickness, 5.5 mm; spacing, 1 mm; matrix 204×320). DWI was completed with a readout-segmented echo-planar imaging (RS-EPI)-DWI sequence (TR/TE, 4,670 ms/63 ms; field of view, 182×240 mm; slice thickness, 3 mm; spacing, 1 mm; matrix 88×116) with identical slice locations to those of transverse T2WI, and b values of 50, 1,000, and 1,500 s/mm2. The apparent diffusion coefficient (ADC) value was calculated using workstation with b values of 50 and 1,000 s/mm2, and an ADC map was also generated.

**Appendix E2**: **Mehralivand grading system**


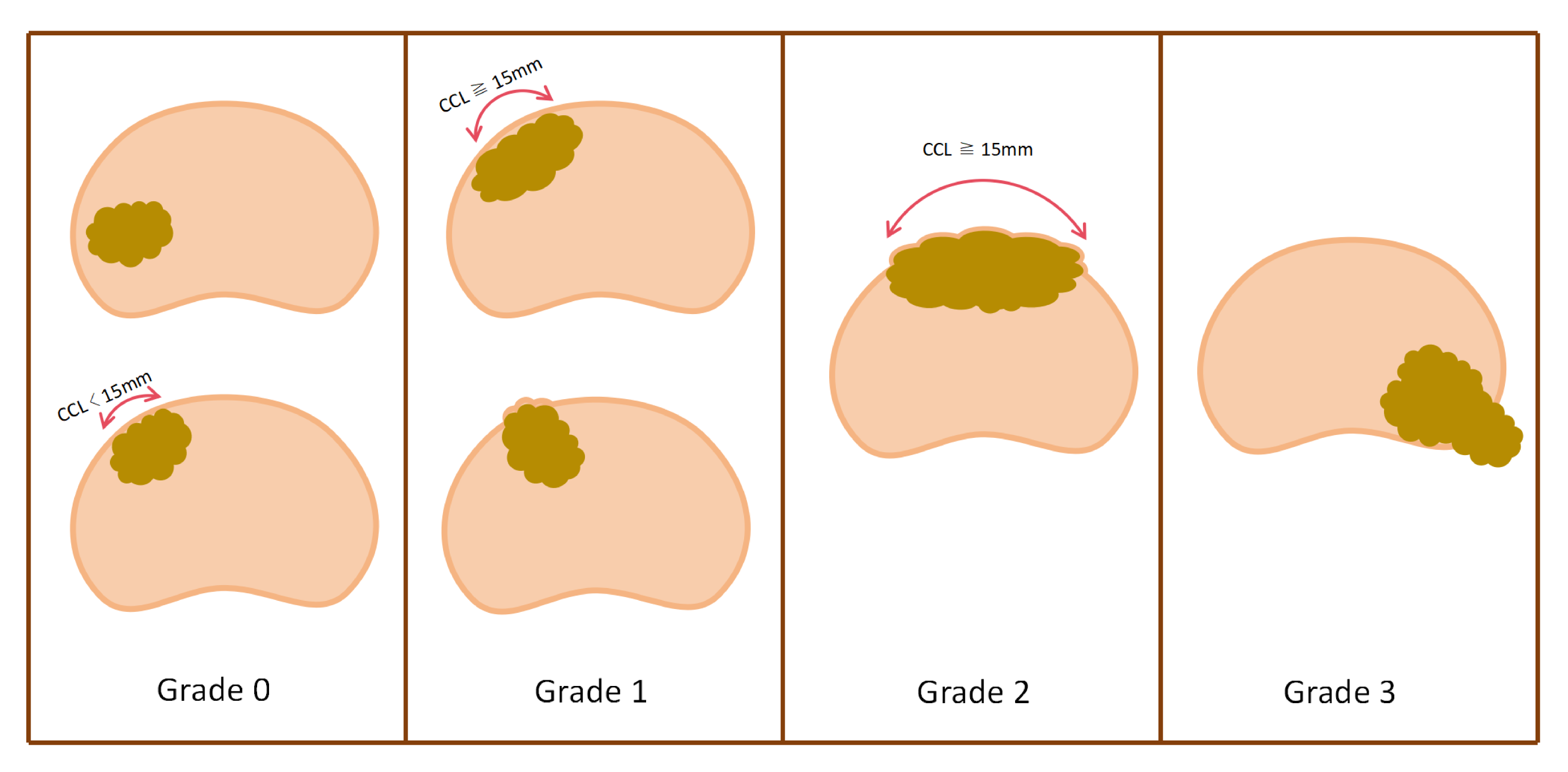


The Mehralivand grading system is primarily based on the curvilinear contact length (CCL) between the lesion and the prostatic capsule, as well as the morphological changes in the prostatic capsule adjacent to the lesion. The criteria are as follows: (1) Grade 0: no significant capsular contact or CCL < 15 mm, considered EPE-negative; (2) Grade 1: CCL ≥ 15 mm, or irregular or bulging capsule, considered EPE-positive; (3) Grade 2: CCL ≥ 15 mm and irregular or bulging capsule, considered EPE-positive; (4) Grade 3: MRI shows clear capsular breach, considered EPE-positive. CCL measurement is performed using the curvilinear measurement module in the ITK-SNAP (v. 4.7.2) software platform.

**Appendix E3**: **PSMA-PET/CT imaging specifics, PSMA-PET/CT image preprocessing, and feature extraction**

**PSMA-PET/CT imaging specifics**

^18^F-PSMA-1007 was produced automatically by cyclotron (Siemens CTI RDS Eclips ST, Knoxville, TN, USA) using ALLINONE synthetic module (Trasis, Belgium) in our center. A PET/CT scan was performed using either a Siemens mCT Flow PET/CT scanner (Siemens CTI RDS Eclips ST, Knoxville, Tennessee, USA) or a United Imaging mCT Flow PET/CT scanner (uMI 780, Shanghai, China). Non-contrast-enhanced CT scans were performed with a 3 mm slice thickness and 2 mm increments, with soft tissue reconstruction at 120 kV. 3D PET scans from the skull base to the knee (matrix 200 × 200) were performed immediately after the CT scan. A multimodal computer platform (Syngo; Siemens Healthcare) was used for image review and processing. The system reconstructed CT, PET, and fused PET/CT images from axial, coronal, and sagittal views, respectively.

**PSMA-PET/CT image preprocessing**

PSMA-PET/CT images were preprocessed through the following steps: (1) Tumor masks were segmented on PET and CT images simultaneously by an experienced nuclear medicine physician (B.L., with > 10 years of experience in FDG-PET/CT), using a semi-automatic segmentation algorithm available in 3D Slicer software (version 4.8.0, http://www.slicer.org). The semi-automatic segmentation masks were then manually adjusted and refined, also using 3D Slicer, by a senior nuclear medicine physician (S.S., with > 15 years of experience in FDG-PET/CT) to ensure reliability of the segmented masks. (2) The raw PET/CT images were covered to value in Hounsfield Unit (HU) by multiplying rescale slope and adding the rescale intercept (3) PET data in HU were normalized based on body mass, injected dose and series time. The derived body mass, injected dose and decay correction (calculated by time) were applied to convert PET images into SUV maps. (4) Normalized PSMA-PET/CT images were resampled into isotropic voxels of unit dimension to ensure comparability, where 1 voxel corresponds to 1 mm3. Specifically, PET/CT images and segmentation masks were resampled via linear interpolation and nearest-neighbor interpolation, respectively. (5) Resampled PET images were first normalized by the SUV bound and then standardized to range [0, 255], while resampled CT images were windowed (also known as grey-level mapping, contrast stretching, histogram modification or contrast enhancement) with a window width of 350 and a level values of 50, and then mapped to range [0, 255]. (6) Alignment of PET, CT, and Label Data: Align the centers of the PET and label data with the CT image. Crop or apply zero-padding to these data as needed to match the dimensions of the CT image. (7) The PET, CT and mask images were cropped into original high×256×256 Regions-of-Interest (ROIs) by fixed-position cropping.

**Feature extraction**

Using Python 3.10.13 and the Pyradiomics package, PET and CT HRFs were obtained from the original images and 8 filter-based images, which included shape features of the original image, first-order texture features, gray level cooccurrence matrix (GLCM), gray level dEPEndence matrix (GLDM), gray level run length matrix (GLRLM), neighboring gray-tone difference matrix (NGTDM) and gray level size zone matrix (GLSZM) of the original image and the eight direved images generated by Daubechies wavelet transform.

**Appendix E4: The specific features retained for each modality.**

**Feature Selection Process:**

1. **T-test:** An independent two-sample T-test was conducted to determine whether the mean and distribution of features between two independent groups exhibited significant differences (significance level: p < 0.05).
2. **Feature Selection Using M3USelector:** After the initial T-test, further feature selection was performed using the M3USelector.

**Comparison of Features Across Different Modalities:**

**Single-modality PET：**

After the first step, 507 features remained, and 20 features were selected after the second step.

Selected features:


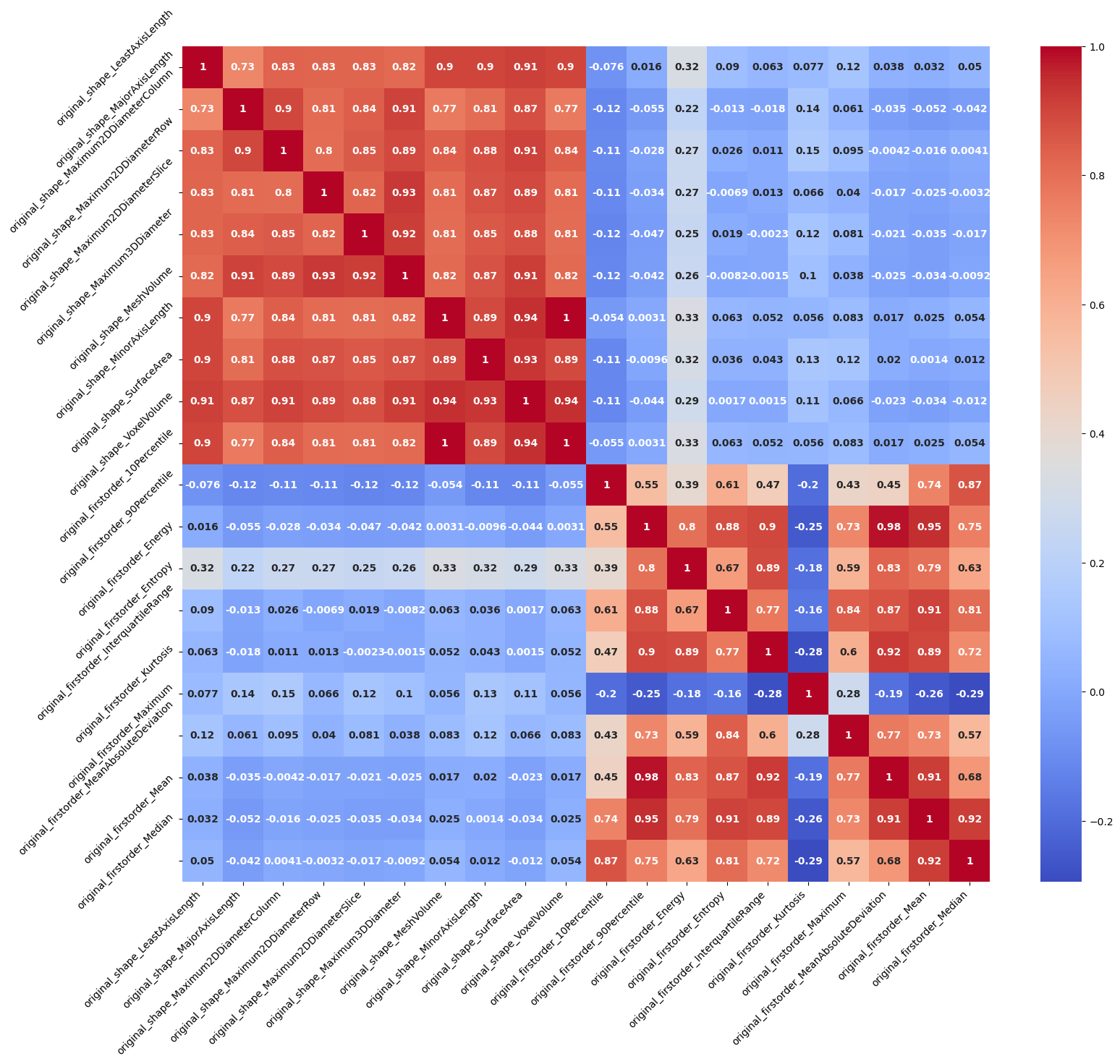


['original_shape_LeastAxisLength',

'original_shape_MajorAxisLength',

'original_shape_Maximum2DDiameterColumn',

'original_shape_Maximum2DDiameterRow',

'original_shape_Maximum2DDiameterSlice',

'original_shape_Maximum3DDiameter',

'original_shape_MeshVolume',

'original_shape_MinorAxisLength',

'original_shape_SurfaceArea',

'original_shape_VoxelVolume',

'original_firstorder_10Percentile',

'original_firstorder_90Percentile',

'original_firstorder_Energy',

'original_firstorder_Entropy',

'original_firstorder_InterquartileRange',

'original_firstorder_Kurtosis',

'original_firstorder_Maximum',

'original_firstorder_MeanAbsoluteDeviation',

'original_firstorder_Mean',

'original_firstorder_Median']

**Single-modality CT:**

After the first step, 207 features remained, and 20 features were selected after the second step.

Selected features:


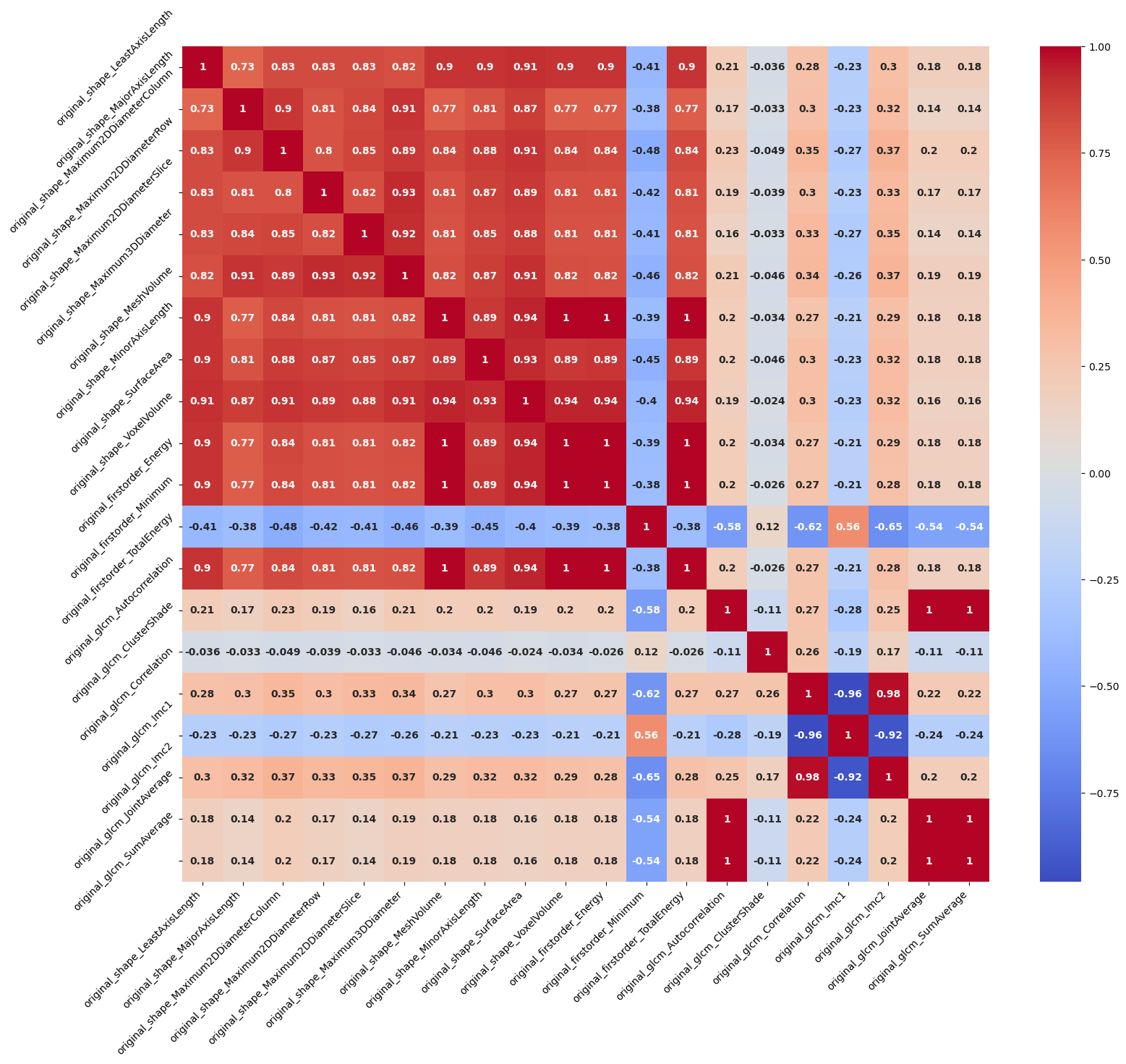


['original_shape_LeastAxisLength',

'original_shape_MajorAxisLength',

'original_shape_Maximum2DDiameterColumn',

'original_shape_Maximum2DDiameterRow',

'original_shape_Maximum2DDiameterSlice',

'original_shape_Maximum3DDiameter',

'original_shape_MeshVolume',

'original_shape_MinorAxisLength',

'original_shape_SurfaceArea',

'original_shape_VoxelVolume',

'original_firstorder_Energy',

'original_firstorder_Minimum',

'original_firstorder_TotalEnergy',

'original_glcm_Autocorrelation',

'original_glcm_ClusterShade',

'original_glcm_Correlation',

'original_glcm_Imc1',

'original_glcm_Imc2',

'original_glcm_JointAverage',

'original_glcm_SumAverage']

**Multi-modality PET concatenate with CT**

After the first step, 714 features (507 from PET + 207 from CT) remained, and 20 features were selected after the second step.

Selected features:


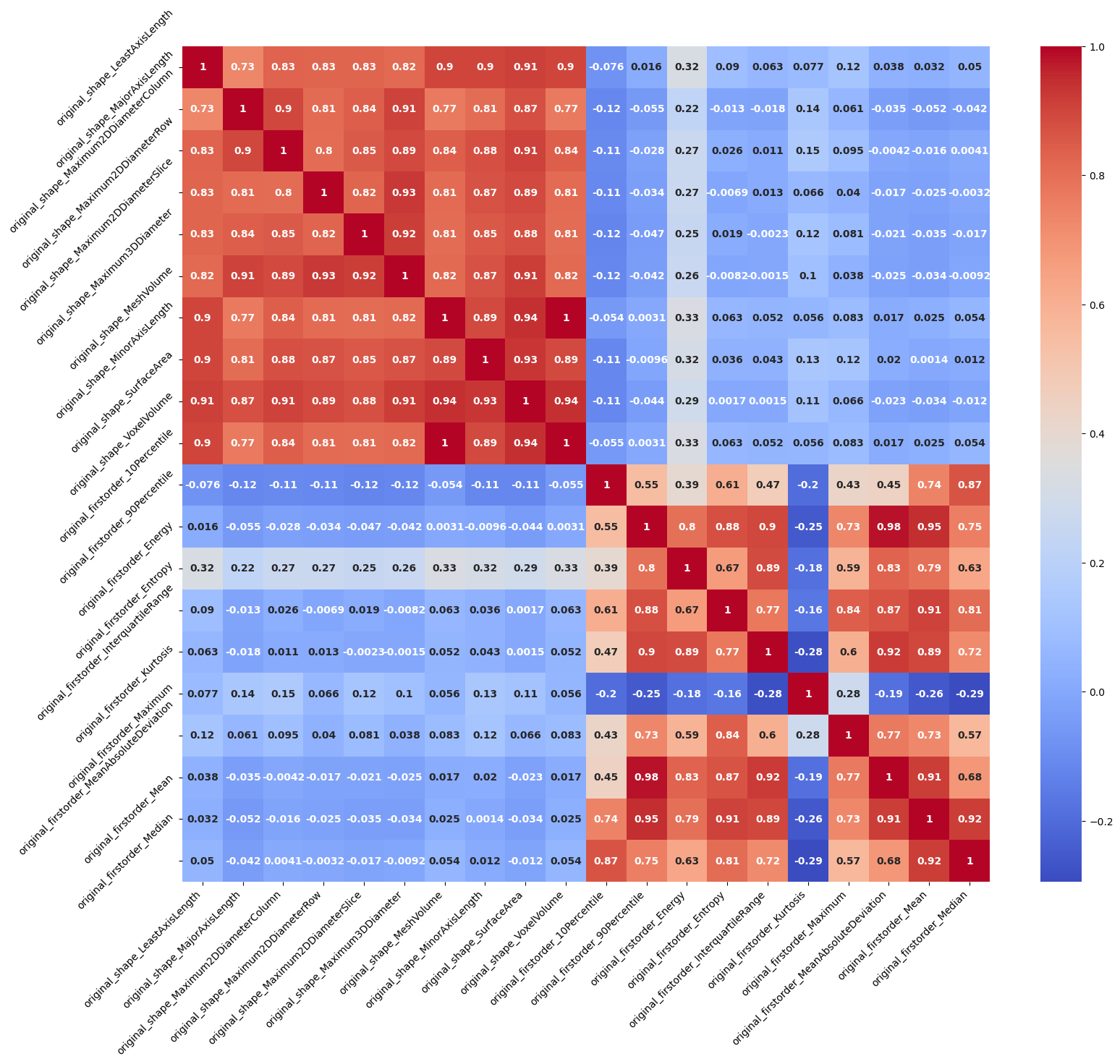


['original_shape_LeastAxisLength',

'original_shape_MajorAxisLength',

'original_shape_Maximum2DDiameterColumn',

'original_shape_Maximum2DDiameterRow',

'original_shape_Maximum2DDiameterSlice',

'original_shape_Maximum3DDiameter',

'original_shape_MeshVolume',

'original_shape_MinorAxisLength',

'original_shape_SurfaceArea',

'original_shape_VoxelVolume',

'original_firstorder_10Percentile',

'original_firstorder_90Percentile',

'original_firstorder_Energy',

'original_firstorder_Entropy',

'original_firstorder_InterquartileRange',

'original_firstorder_Kurtosis',

'original_firstorder_Maximum',

'original_firstorder_MeanAbsoluteDeviation',

'original_firstorder_Mean',

'original_firstorder_Median']

**Multi-modality PET concatenate with CT**

After the first step, 366 features remained, and 20 features were selected after the second step.

Selected features:


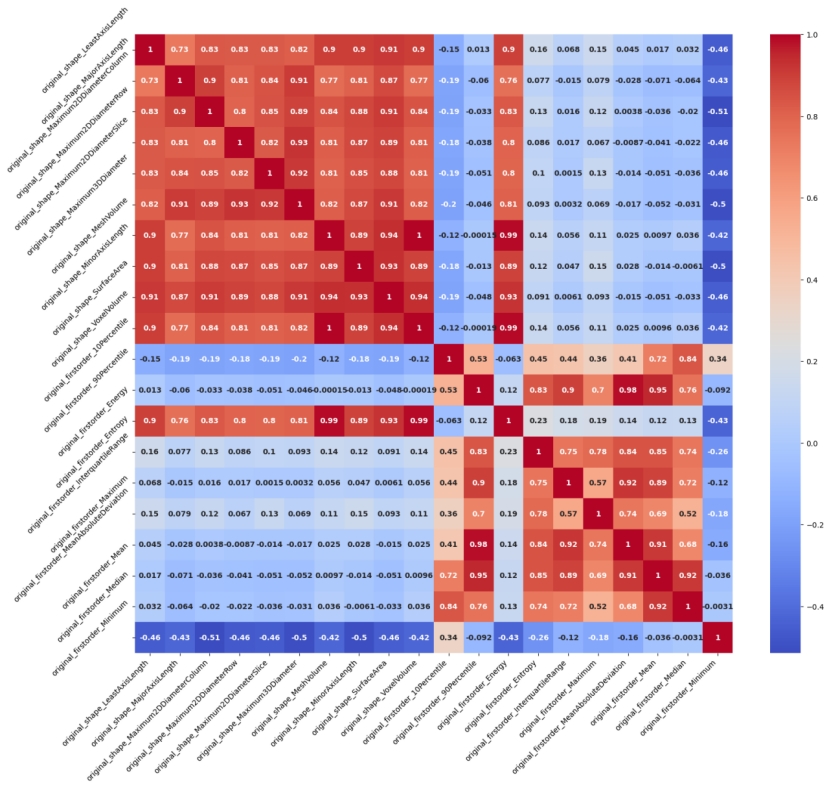


['original_shape_LeastAxisLength',

'original_shape_MajorAxisLength',

'original_shape_Maximum2DDiameterColumn',

'original_shape_Maximum2DDiameterRow',

'original_shape_Maximum2DDiameterSlice',

'original_shape_Maximum3DDiameter',

'original_shape_MeshVolume',

'original_shape_MinorAxisLength',

'original_shape_SurfaceArea',

'original_shape_VoxelVolume',

'original_firstorder_10Percentile',

'original_firstorder_90Percentile',

'original_firstorder_Energy',

'original_firstorder_Entropy',

'original_firstorder_InterquartileRange',

'original_firstorder_Maximum',

'original_firstorder_MeanAbsoluteDeviation',

'original_firstorder_Mean',

'original_firstorder_Median',

'original_firstorder_Minimum']

**Appendix E5: The specific features retained for each modality.**

1. **Single-modality PET**

Before Feature Selection

| SVM | Round 1 | Round 2 | Round 3 | Round 4 | Round 5 | Mean |
| --- | --- | --- | --- | --- | --- | --- |
| Accuracy | 0.59 | 0.71 | 0.61 | 0.66 | 0.76 | 0.67 |
| AUC | 0.63 | 0.79 | 0.68 | 0.74 | 0.86 | 0.74 |
| NPV | 0.62 | 0.68 | 0.62 | 0.65 | 0.72 | 0.66 |
| PPV | 0.50 | 0.86 | 0.57 | 0.70 | 0.83 | 0.69 |
| Specificity | 0.75 | 0.96 | 0.88 | 0.87 | 0.91 | 0.87 |
| Sensitivity | 0.35 | 0.35 | 0.24 | 0.39 | 0.56 | 0.38 |

| Random Forest | Round 1 | Round 2 | Round 3 | Round 4 | Round 5 | Mean |
| --- | --- | --- | --- | --- | --- | --- |
| Accuracy | 0.61 | 0.68 | 0.61 | 0.68 | 0.71 | 0.66 |
| AUC | 0.67 | 0.76 | 0.70 | 0.77 | 0.79 | 0.74 |
| NPV | 0.64 | 0.70 | 0.64 | 0.69 | 0.69 | 0.67 |
| PPV | 0.54 | 0.64 | 0.54 | 0.67 | 0.75 | 0.63 |
| Specificity | 0.75 | 0.79 | 0.75 | 0.78 | 0.87 | 0.79 |
| Sensitivity | 0.41 | 0.53 | 0.41 | 0.56 | 0.5 | 0.48 |


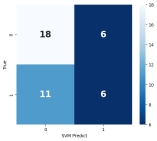

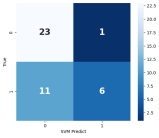

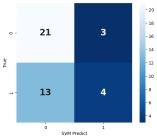

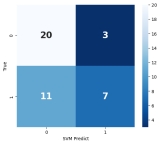

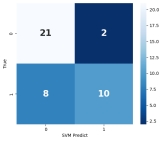


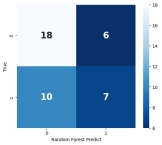

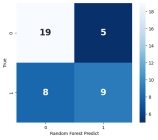

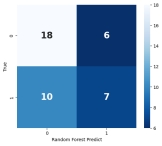

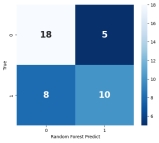

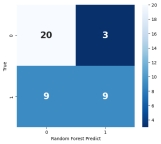


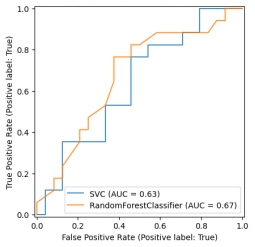

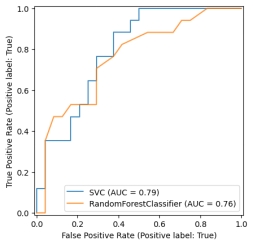

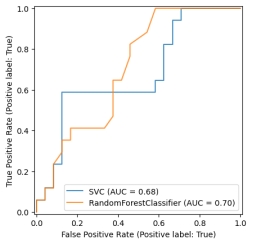

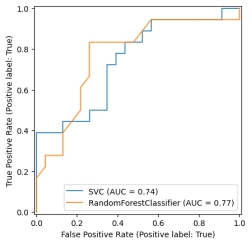

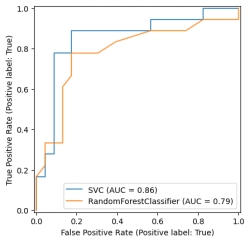


After Feature Selection

| SVM | Round 1 | Round 2 | Round 3 | Round 4 | Round 5 | Mean |
| --- | --- | --- | --- | --- | --- | --- |
| Accuracy | 0.66 | 0.71 | 0.63 | 0.66 | 0.73 | 0.68 |
| AUC | 0.62 | 0.80 | 0.61 | 0.76 | 0.82 | 0.72 |
| NPV | 0.66 | 0.68 | 0.64 | 0.66 | 0.7 | 0.67 |
| PPV | 0.67 | 0.86 | 0.62 | 0.67 | 0.82 | 0.73 |
| Specificity | 0.88 | 0.96 | 0.88 | 0.83 | 0.91 | 0.89 |
| Sensitivity | 0.35 | 0.35 | 0.29 | 0.44 | 0.5 | 0.39 |

| Random Forest | Round 1 | Round 2 | Round 3 | Round 4 | Round 5 | Mean |
| --- | --- | --- | --- | --- | --- | --- |
| Accuracy | 0.63 | 0.76 | 0.61 | 0.73 | 0.83 | 0.71 |
| AUC | 0.65 | 0.80 | 0.64 | 0.82 | 0.83 | 0.75 |
| NPV | 0.67 | 0.79 | 0.65 | 0.73 | 0.79 | 0.73 |
| PPV | 0.57 | 0.71 | 0.53 | 0.73 | 0.92 | 0.69 |
| Specificity | 0.75 | 0.79 | 0.71 | 0.83 | 0.96 | 0.81 |
| Sensitivity | 0.47 | 0.71 | 0.47 | 0.61 | 0.67 | 0.59 |


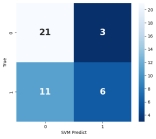

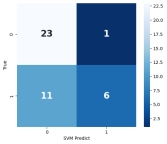

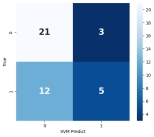

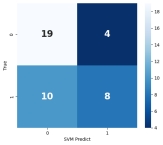

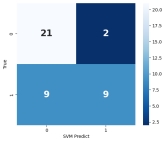

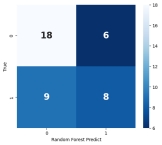

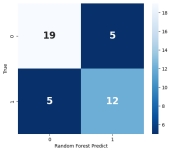

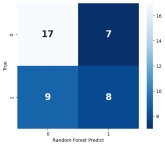

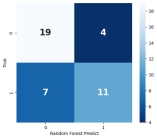

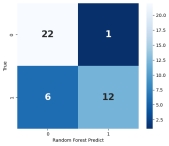


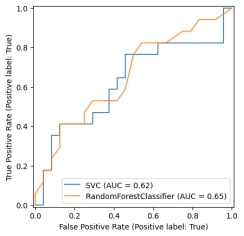

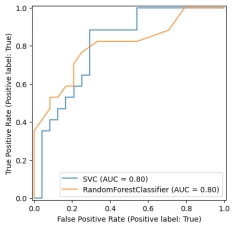

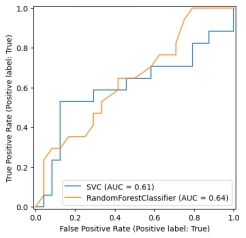


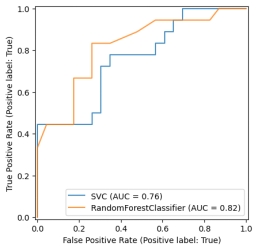

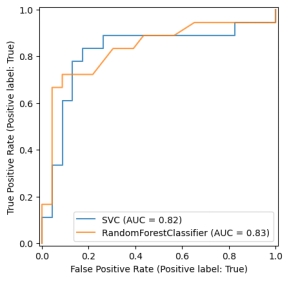


1. **Single-modality CT**

Before Feature Selection

| SVM | Round 1 | Round 2 | Round 3 | Round 4 | Round 5 | Mean |
| --- | --- | --- | --- | --- | --- | --- |
| Accuracy | 0.59 | 0.63 | 0.68 | 0.56 | 0.61 | 0.61 |
| AUC | 0.50 | 0.66 | 0.51 | 0.56 | 0.71 | 0.59 |
| NPV | 0.60 | 0.62 | 0.65 | 0.56 | 0.59 | 0.60 |
| PPV | 0.50 | 1.00 | 1.00 | 0.00 | 1.00 | 0.70 |
| Specificity | 0.88 | 1.00 | 1.00 | 1.00 | 1.00 | 0.98 |
| Sensitivity | 0.18 | 0.12 | 0.24 | 0.00 | 0.11 | 0.13 |

| Random Forest | Round 1 | Round 2 | Round 3 | Round 4 | Round 5 | Mean |
| --- | --- | --- | --- | --- | --- | --- |
| Accuracy | 0.49 | 0.66 | 0.44 | 0.61 | 0.46 | 0.53 |
| AUC | 0.52 | 0.72 | 0.42 | 0.57 | 0.42 | 0.53 |
| NPV | 0.56 | 0.67 | 0.52 | 0.59 | 0.52 | 0.57 |
| PPV | 0.36 | 0.64 | 0.31 | 0.75 | 0.38 | 0.49 |
| Specificity | 0.62 | 0.83 | 0.54 | 0.96 | 0.57 | 0.70 |
| Sensitivity | 0.29 | 0.41 | 0.29 | 0.17 | 0.33 | 0.30 |


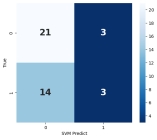

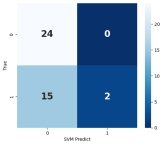

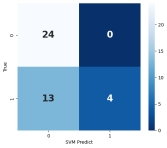

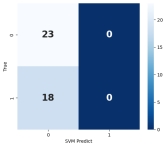

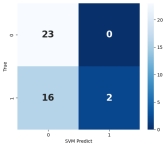


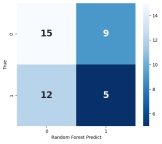

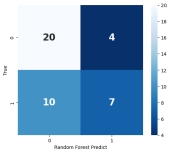

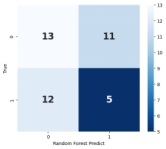

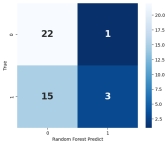

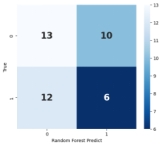


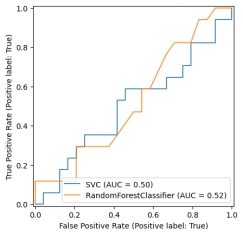

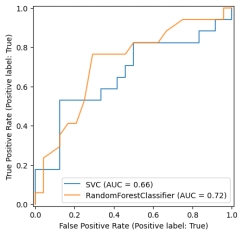

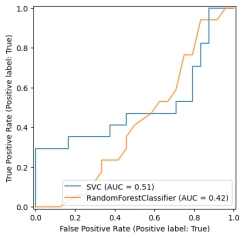


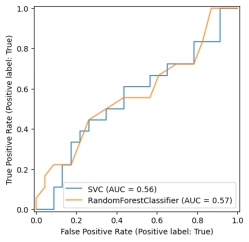

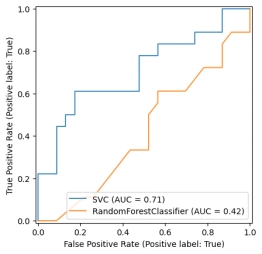


After Feature Selection

| SVM | Round 1 | Round 2 | Round 3 | Round 4 | Round 5 | Mean |
| --- | --- | --- | --- | --- | --- | --- |
| Accuracy | 0.59 | 0.63 | 0.68 | 0.56 | 0.63 | 0.62 |
| AUC | 0.52 | 0.70 | 0.51 | 0.53 | 0.78 | 0.61 |
| NPV | 0.60 | 0.62 | 0.65 | 0.56 | 0.61 | 0.61 |
| PPV | 0.50 | 1.00 | 1.00 | 0.00 | 1.00 | 0.70 |
| Specificity | 0.88 | 1.00 | 1.00 | 1.00 | 1.00 | 0.98 |
| Sensitivity | 0.18 | 0.12 | 0.24 | 0.00 | 0.17 | 0.14 |

| Random Forest | Round 1 | Round 2 | Round 3 | Round 4 | Round 5 | Mean |
| --- | --- | --- | --- | --- | --- | --- |
| Accuracy | 0.56 | 0.59 | 0.51 | 0.68 | 0.61 | 0.59 |
| AUC | 0.57 | 0.69 | 0.40 | 0.67 | 0.70 | 0.61 |
| NPV | 0.62 | 0.62 | 0.57 | 0.67 | 0.63 | 0.62 |
| PPV | 0.47 | 0.5 | 0.38 | 0.73 | 0.57 | 0.53 |
| Specificity | 0.67 | 0.75 | 0.67 | 0.87 | 0.74 | 0.74 |
| Sensitivity | 0.41 | 0.35 | 0.29 | 0.44 | 0.44 | 0.39 |


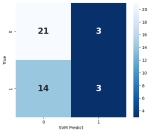

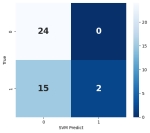

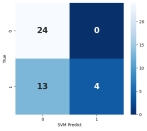

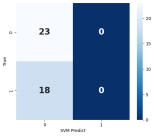

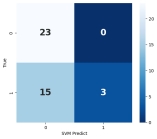

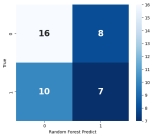

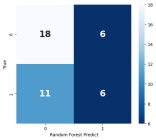

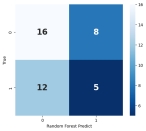

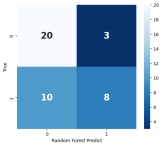

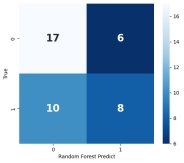


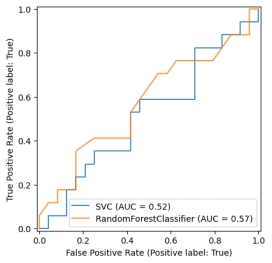

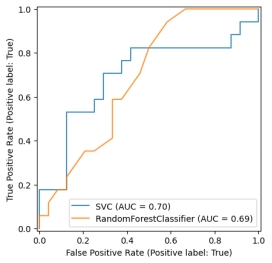

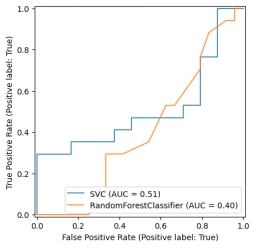

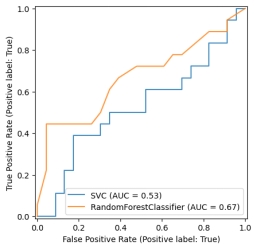

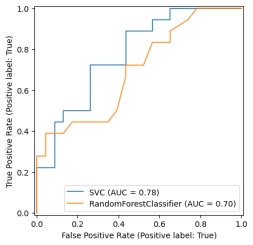


1. **Multi-modality PET concatenate CT**

Before Feature Selection

| SVM | Round 1 | Round 2 | Round 3 | Round 4 | Round 5 | Mean |
| --- | --- | --- | --- | --- | --- | --- |
| Accuracy | 0.63 | 0.63 | 0.63 | 0.61 | 0.63 | 0.63 |
| AUC | 0.56 | 0.76 | 0.57 | 0.68 | 0.86 | 0.69 |
| NPV | 0.64 | 0.62 | 0.62 | 0.59 | 0.61 | 0.62 |
| PPV | 0.62 | 1.00 | 0.75 | 1.00 | 1.00 | 0.87 |
| Specificity | 0.88 | 1.00 | 0.96 | 1.00 | 1.00 | 0.97 |
| Sensitivity | 0.29 | 0.12 | 0.18 | 0.11 | 0.17 | 0.17 |

| Random Forest | Round 1 | Round 2 | Round 3 | Round 4 | Round 5 | Mean |
| --- | --- | --- | --- | --- | --- | --- |
| Accuracy | 0.61 | 0.71 | 0.66 | 0.73 | 0.66 | 0.67 |
| AUC | 0.64 | 0.78 | 0.66 | 0.77 | 0.79 | 0.73 |
| NPV | 0.64 | 0.71 | 0.69 | 0.71 | 0.65 | 0.68 |
| PPV | 0.54 | 0.69 | 0.60 | 0.77 | 0.70 | 0.66 |
| Specificity | 0.75 | 0.83 | 0.75 | 0.87 | 0.87 | 0.81 |
| Sensitivity | 0.41 | 0.53 | 0.53 | 0.56 | 0.39 | 0.48 |


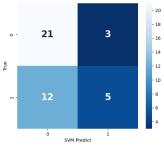

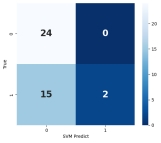

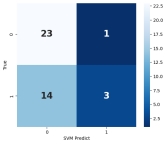

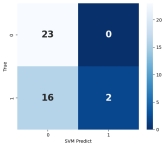

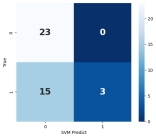

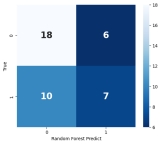

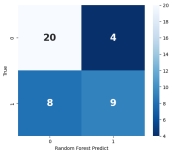

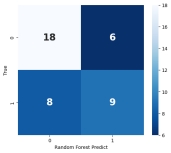

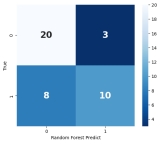

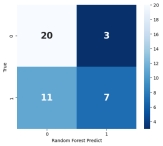


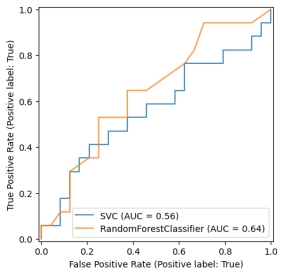

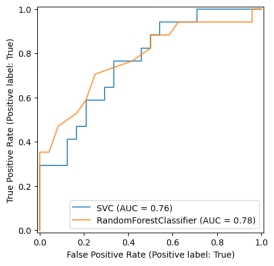

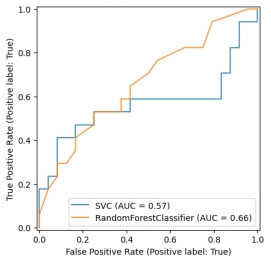

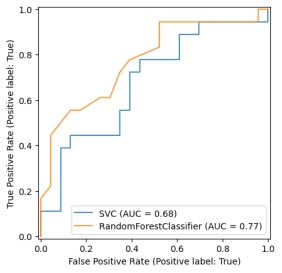

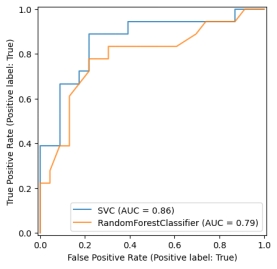


After Feature Selection

| SVM | Round 1 | Round 2 | Round 3 | Round 4 | Round 5 | Mean |
| --- | --- | --- | --- | --- | --- | --- |
| Accuracy | 0.66 | 0.71 | 0.63 | 0.66 | 0.73 | 0.68 |
| AUC | 0.62 | 0.80 | 0.61 | 0.76 | 0.82 | 0.72 |
| NPV | 0.66 | 0.68 | 0.64 | 0.66 | 0.7 | 0.67 |
| PPV | 0.67 | 0.86 | 0.62 | 0.67 | 0.82 | 0.73 |
| Specificity | 0.88 | 0.96 | 0.88 | 0.83 | 0.91 | 0.89 |
| Sensitivity | 0.35 | 0.35 | 0.29 | 0.44 | 0.5 | 0.39 |

| Random Forest | Round 1 | Round 2 | Round 3 | Round 4 | Round 5 | Mean |
| --- | --- | --- | --- | --- | --- | --- |
| Accuracy | 0.63 | 0.76 | 0.61 | 0.73 | 0.83 | 0.71 |
| AUC | 0.65 | 0.80 | 0.64 | 0.82 | 0.83 | 0.75 |
| NPV | 0.67 | 0.79 | 0.65 | 0.73 | 0.79 | 0.73 |
| PPV | 0.57 | 0.71 | 0.53 | 0.73 | 0.92 | 0.69 |
| Specificity | 0.75 | 0.79 | 0.71 | 0.83 | 0.96 | 0.81 |
| Sensitivity | 0.47 | 0.71 | 0.47 | 0.61 | 0.67 | 0.59 |


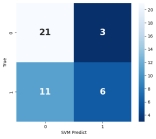

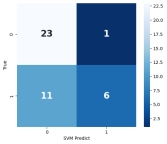

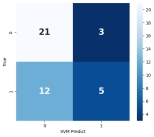

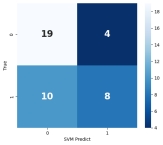

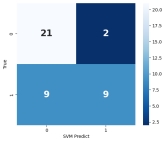

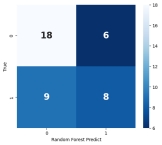

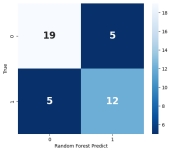

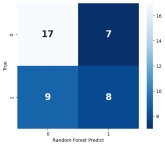

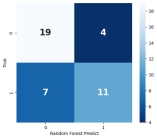

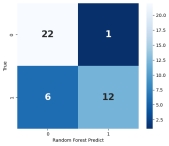


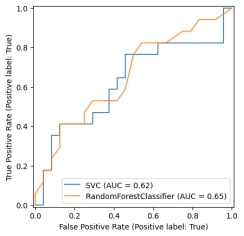

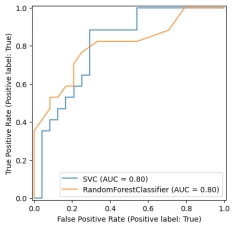

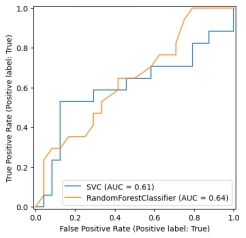


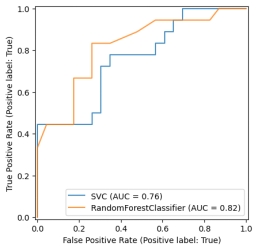

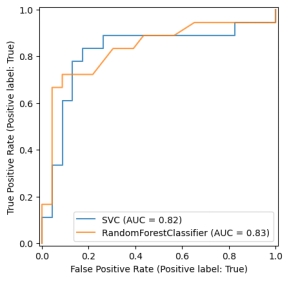


1. **Multi-modality PET sum CT average**

Before Feature Selection

| SVM | Round 1 | Round 2 | Round 3 | Round 4 | Round 5 | Mean |
| --- | --- | --- | --- | --- | --- | --- |
| Accuracy | 0.59 | 0.63 | 0.59 | 0.59 | 0.63 | 0.61 |
| AUC | 0.53 | 0.75 | 0.53 | 0.66 | 0.79 | 0.65 |
| NPV | 0.61 | 0.62 | 0.62 | 0.57 | 0.61 | 0.61 |
| PPV | 0.50 | 1.00 | 0.50 | 1.00 | 1.00 | 0.80 |
| Specificity | 0.79 | 1.00 | 0.75 | 1.00 | 1.00 | 0.91 |
| Sensitivity | 0.37 | 0.12 | 0.35 | 0.06 | 0.17 | 0.21 |

| Random Forest | Round 1 | Round 2 | Round 3 | Round 4 | Round 5 | Mean |
| --- | --- | --- | --- | --- | --- | --- |
| Accuracy | 0.63 | 0.73 | 0.68 | 0.63 | 0.68 | 0.67 |
| AUC | 0.69 | 0.77 | 0.66 | 0.72 | 0.74 | 0.72 |
| NPV | 0.67 | 0.74 | 0.69 | 0.67 | 0.67 | 0.69 |
| PPV | 0.57 | 0.71 | 0.67 | 0.59 | 0.73 | 0.65 |
| Specificity | 0.75 | 0.83 | 0.83 | 0.7 | 0.87 | 0.80 |
| Sensitivity | 0.47 | 0.59 | 0.47 | 0.56 | 0.44 | 0.51 |


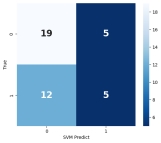

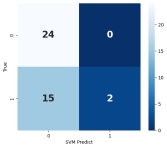

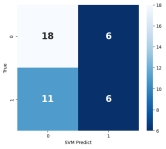

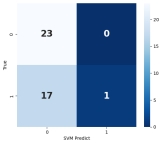

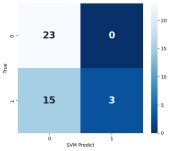


After Feature Selection

| SVM | Round 1 | Round 2 | Round 3 | Round 4 | Round 5 | Mean |
| --- | --- | --- | --- | --- | --- | --- |
| Accuracy | 0.59 | 0.63 | 0.59 | 0.59 | 0.63 | 0.61 |
| AUC | 0.52 | 0.71 | 0.54 | 0.64 | 0.64 | 0.61 |
| NPV | 0.61 | 0.62 | 0.62 | 0.57 | 0.61 | 0.61 |
| PPV | 0.50 | 1.00 | 0.50 | 1.00 | 1.00 | 0.80 |
| Specificity | 0.79 | 1.00 | 0.75 | 1.00 | 1.00 | 0.91 |
| Sensitivity | 0.29 | 0.12 | 0.35 | 0.06 | 0.17 | 0.20 |

| Random Forest | Round 1 | Round 2 | Round 3 | Round 4 | Round 5 | Mean |
| --- | --- | --- | --- | --- | --- | --- |
| Accuracy | 0.63 | 0.76 | 0.63 | 0.78 | 0.76 | 0.71 |
| AUC | 0.69 | 0.79 | 0.68 | 0.84 | 0.83 | 0.77 |
| NPV | 0.67 | 0.79 | 0.67 | 0.77 | 0.74 | 0.73 |
| PPV | 0.57 | 0.71 | 0.57 | 0.80 | 0.79 | 0.69 |
| Specificity | 0.75 | 0.79 | 0.75 | 0.87 | 0.87 | 0.81 |
| Sensitivity | 0.47 | 0.71 | 0.47 | 0.67 | 0.61 | 0.59 |

**Table S1** Characteristics of patients

| Clinical features |  |
| --- | --- |
| Age , years,  median (IQR) | 70 (61 - 74) |
| BMI, kg/m², median (IQR) | 24.5 (22.2 - 26.1) |
| PSA at PET,  ng/mL, median (IQR) | 14.4 (6.9 - 29.7) |
| Biopsy ISUP grade group, n (%) |  |
| ISUP 1 | 13 (11.0%) |
| ISUP 2 | 36 (30.5%) |
| ISUP 3 | 35 (29.7%) |
| ISUP 4 | 26 (22.0%) |
| ISUP 5 | 8 (6.8%) |
| Positive biopsies, %,  median (IQR) | 58.3 %  (45.9%- 70.9% ) |

IQR = interquartile range; PSA = prostate-specific antigen; PET = positron emission tomography;

ISUP = International Society of Urological Pathology.

**Table S2** PPV for Diagnosing EPE Across Different Mehralivand Grades

| Mehralivand Grading System | PPV (%) |
| --- | --- |
| Reader 1 |  |
| 0 | 73.6% [28/38] |
| 1 | 47.1% [8/17] |
| 2 | 85.7% [6/7] |
| 3 | 100% [1/1] |
| Reader 2 |  |
| 0 | 71.1% [27/38] |
| 1 | 44.4% [8/18] |
| 2 | 83.3% [5/6] |
| 3 | 100% [1/1] |
| Reader 3 |  |
| 0 | 68.4% [26/38] |
| 1 | 42.1% [8/19] |
| 2 | 80.0% [4/5] |
| 3 | 100% [1/1] |

Note. PPV = positive predictive value.
